# Photo-induced electric polarizability of Fe_3_O_4_ nanoparticles in weak optical fields

**DOI:** 10.1186/1556-276X-8-317

**Published:** 2013-07-09

**Authors:** Valentin A Milichko, Anton I Nechaev, Viktor A Valtsifer, Vladimir N Strelnikov, Yurii N Kulchin, Vladimir P Dzyuba

**Affiliations:** 1 Institute of Automation and Control Processes, FEB RAS, Radio 5, Vladivostok 690041, Russia; 2 Far Eastern Federal University, Sukhanova 8, Vladivostok 690950, Russia; 3 Institute of Technical Chemistry, UB RAS, Academician Korolyov 3, Perm 614013, Russia

**Keywords:** Magnetite nanoparticles, Electric polarizability, Low-intensity visible radiation

## Abstract

Using a developed co-precipitation method, we synthesized spherical Fe_3_O_4_ nanoparticles with a wide nonlinear absorption band of visible radiation. Optical properties of the synthesized nanoparticles dispersed in an optically transparent copolymer of methyl methacrylate with styrene were studied by optical spectroscopy and z-scan techniques. We found that the electric polarizability of Fe_3_O_4_ nanoparticles is altered by low-intensity visible radiation (*I* ≤ 0.2 kW/cm^2^; λ = 442 and 561 nm) and reaches a value of 10^7^ Å^3^. The change in polarizability is induced by the intraband phototransition of charge carriers. This optical effect may be employed to improve the drug uptake properties of Fe_3_O_4_ nanoparticles.

**PACS:**

33.15.Kr

78.67.Bf

42.70.Nq

## Background

Magnetite (FeO*Fe_2_O_3_, or Fe_3_O_4_) nanoparticles, and materials based on them, have been successfully used to solve applied problems in biology and magneto-optics. Pronounced superparamagnetic [1-4] and ferromagnetic [5] properties at room temperature enable the use of these nanoparticles in magnetic resonance imaging [6-9] and biosensing [9] as well as in drug delivery and drug uptake applications [8-13]. Because they possess magneto-optical properties [14,15], Fe_3_O_4_ nanoparticles have also been used to develop tunable filters [16,17] and optical switches [18,19] that operate under magnetic fields.

In fact, Fe_3_O_4_ nanoparticles have been examined for the presence of unique magnetic properties because magnetite is a narrow-gap semiconductor [20-22] and the optical properties of other semiconductor nanoparticles have been thoroughly studied. Currently, there are several experimental and theoretical works dedicated to studying the optical properties of both bulk magnetite [23-26] and its nanoparticles [27-29]. However, some specific optical properties of Fe_3_O_4_ nanoparticles (in particular, the effects of electric polarizability on their biological activity, conductivity, ferroelectricity, and electro-optical properties) as well as the nature of these properties remain virtually unexplored.

In this paper, we demonstrate that Fe_3_O_4_ nanoparticles exhibiting a wide nonlinear absorption band of visible radiation (1.7:3.7 eV) are able to significantly change their electric polarizability when exposed to low-intensity visible radiation (*I* ≤ 0.2 kW/cm^2^). The observed change in polarizability was induced by the intraband phototransition of nanoparticle charge carriers, and polarizability changes were orders of magnitude greater than those of semiconductor nanoparticles and molecules [30,31].

## Experiments

### Synthesis of nanoparticles

There are several techniques for the synthesis of Fe_3_O_4_ nanoparticles with an arbitrary shape and size and for their dispersal in different matrices [4,5,11,12,27,29,32-36]. In this study, we synthesized nanoparticles using co-precipitation method [1,2,13-15,37,38], dispersed them in monomeric methyl methacrylate with styrene (MMAS), and polymerized this composition using pre-polymerization method.

**Figure 1:**
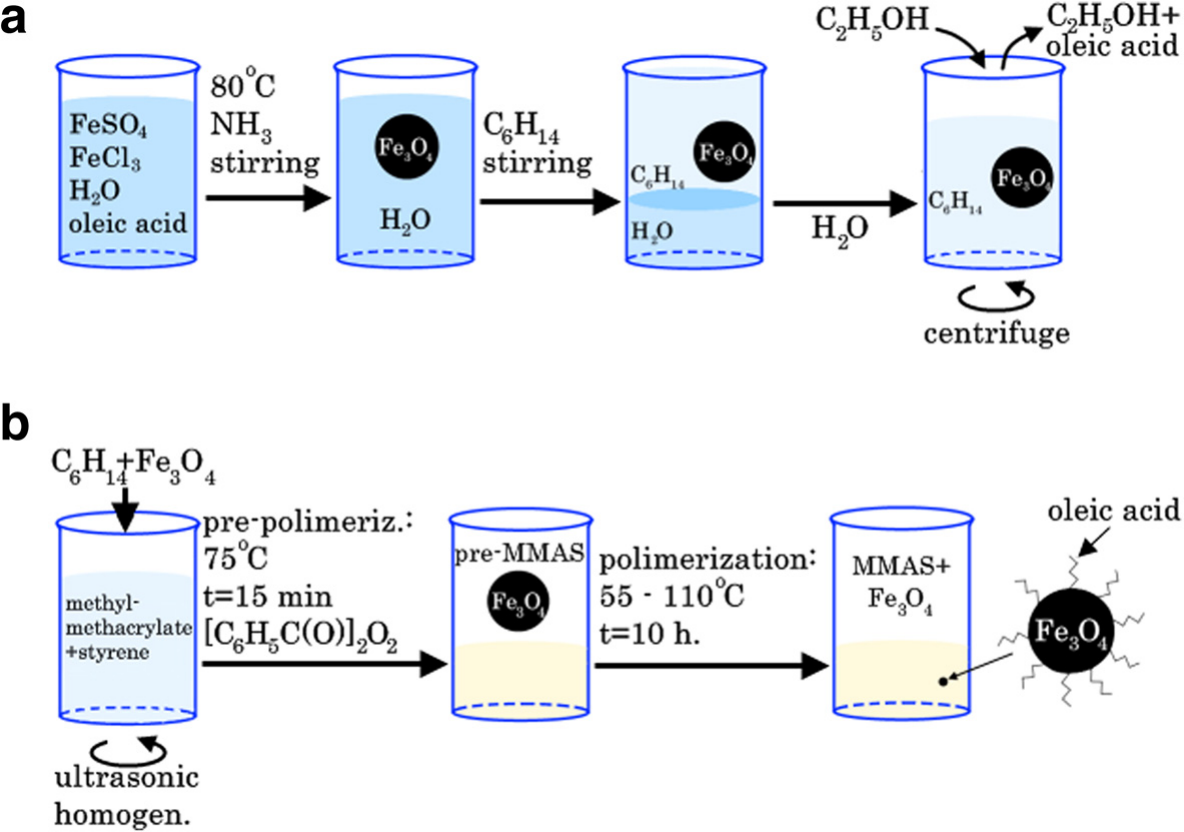
**The developed co-precipitation method. (a)** The synthesis of Fe_3_O_4_ nanoparticles with a monolayer of oleic acid by the developed co-precipitation method and **(b)** the composite MMAS + Fe_3_O_4_ preparation.

In the first step (Figure 1a), Fe_3_O_4_ nanoparticles were synthesized by co-precipitation of soluble salts of ferrous and ferric ions with an aqueous ammonia solution: FeSO_4_*7H_2_O + 2FeCl_3_*6H_2_O + 8NH_3_*H_2_O ↔ Fe_3_O_4_ + 6NH_4_Cl + (NH_4_)_2_SO_4_ + 20H_2_O.

Oleic acid (in a mass ratio of 0.7:1 with the formed Fe_3_O_4_) was added to a 0.5% solution of iron salts (FeSO_4_/FeCl_3_ = 1:2.2 molar ratio) in 0.1 M HCl. The aqueous solution of iron salts was heated to 80°C, followed by the addition of concentrated aqueous ammonia (20% excess). The solution was heated and stirred for an hour.

Stabilized nanoparticles were then extracted from the aqueous phase into a nonpolar organic solvent hexane at a ratio of 1:1. The organic layer containing the iron oxide Fe_3_O_4_ was separated from the aqueous medium. The sample was centrifuged for 15 min (6,000 rpm) to remove larger particles. Excess acid was removed with ethanol.

**Figure 2:**
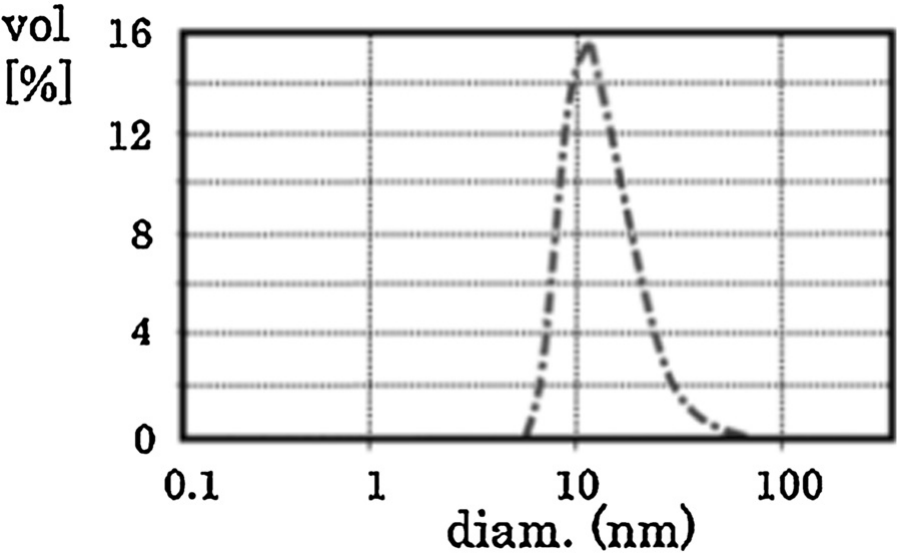
**Nanoparticle size.** The average hydrodynamic diameter of the synthesized nanoparticles (15 nm) dispersed in hexane was determined by dynamic light scattering method (Zetasizer Nano ZS, Malvern, UK) at a laser wavelength of 532 nm.

The size of the nanoparticles was determined by dynamic light scattering method (Zetasizer Nano ZS, Malvern, UK). Measurements were conducted in hexane with a laser wavelength of 532 nm. The average hydrodynamic diameter of the synthesized nanoparticles was 15 nm, as illustrated in Figure 2.

### Composite preparation

The second step (Figure 1b) focused on obtaining a solid composite based on Fe_3_O_4_ nanoparticles and MMAS. The organic solvent containing nanoparticles and monomers (methyl methacrylate with styrene) was subjected to stirring and ultrasonic homogenization. To prevent nanoparticle aggregation during the polymerization process, we used the pre-polymerization method at 75°C because the nanoparticles had different affinities to the monomer and polymer.

Finally, the composite was synthesized *in situ* by radical polymerization. The polymerization of methyl methacrylate with styrene (in the mass ratio of 20:1) proceeded for over 10 h (in a temperature gradient mode that progressed from 55°C to 110°C) in the presence of benzoyl peroxide (10^−3^ mol/L).

The obtained solid composites had 0.001%, 0.003%, 0.005%, and 0.01% volume concentrations of Fe_3_O_4_ nanoparticles in MMAS. Importantly, the synthesized Fe_3_O_4_ nanoparticles generally had a thick layer of acids [36,39] surrounding them to prevent aggregation of the nanoparticle. In our case, the synthesized Fe_3_O_4_ nanoparticles had a monolayer of oleic acid that allowed the nanoparticles to exhibit their specific optical properties.

### UV–vis spectroscopy

**Figure 3:**
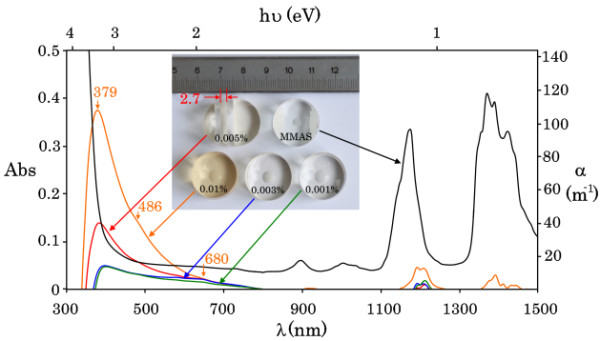
**Absorbance spectra for the MMAS and Fe_3_O_4_ nanoparticle array.** The optical absorbance spectra for pure MMAS and Fe_3_O_4_ nanoparticle arrays with 0.001%, 0.003%, 0.005%, and 0.01% volume concentrations.

Room-temperature optical absorbance spectra of pure MMAS (Figure 3, black curve) and of the composites were obtained using a Varian Cary 5000I spectrophotometer (Agilent Technologies, Santa Clara, CA, USA) over the wavelength range of 300 to 1,500 nm. These spectra allowed the derivation of the absorbance spectra for Fe_3_O_4_ nanoparticle arrays (Figure 3, color curves). Figure 3 shows the absorbance values (Abs) and the absorption coefficients (*α* = (Abs × ln 10)/*l*, where *l* = 7.95 mm is the length of the composite) measured at a maximum radiation intensity of 1 μW/cm^2^.

### z-Scan experiments

Because they have absorption bands of 380 to 650 nm, Fe_3_O_4_ nanoparticles should exhibit an optical response upon external radiation with wavelengths in this band [40]. To detect the optical response of the nanoparticles contained in the composite (0.005% nanoparticle volume concentration), we used the standard z-scan technique [41]. This technique enabled the analysis of changes in the absorption coefficient Δ*α*(*I*) and refractive index Δ*n*(*I*) of the composite and pure MMAS, which were induced by weak optical radiation with different intensities 0 to 0.14 kW/cm^2^.

**Figure 4:**
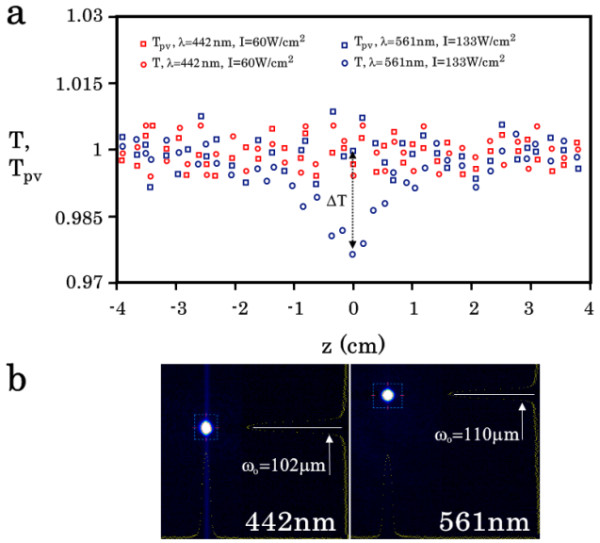
**z-Scan results for the MMAS. (a)** Curves for z-scans with open (circle) *T*(*I*) and closed (square) *T*_pv_(*I*) apertures at radiation wavelengths of 442 nm (red points, 60 W/cm^2^) and 561 nm (blue points, 133 W/cm^2^) for the MMAS sample (*L* = 2.7 mm). **(b)** Profilometer images for the beam waists *ω*_0_.

**Figure 5:**
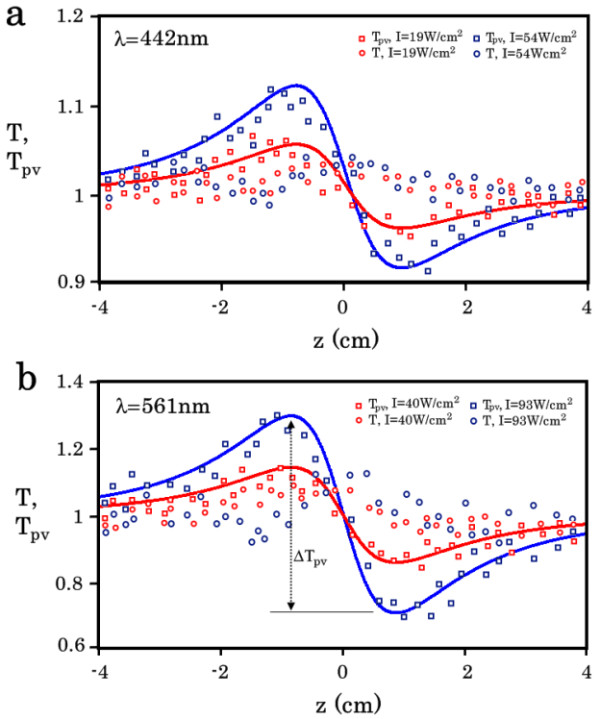
**z-Scan results for the composite.** Curves for z-scans with open (circle) *T*(*I*) and closed (square) *T*_pv_(*I*) apertures at radiation wavelengths of 442 nm **(a)** (red points, 19 W/cm^2^; blue points, 54 W/cm^2^) and 561 nm **(b)** (red points, 40 W/cm^2^; blue points, 93 W/cm^2^) for the composite sample (*L* = 2.7 mm) containing Fe_3_O_4_ nanoparticle with a 0.005% volume concentration.

For radiation sources, we used semiconductor lasers of continuous wave (cw) radiation with wavelengths of 442 nm (blue) and 561 nm (yellow) providing maximal intensities of 0.07 and 0.14 kW/cm^2^. Lenses with focal lengths of 75 mm provided the beam waists *ω*_0_ = 102 and 110 μm for blue and yellow radiation (Figure 4b). The length (*L*) of experimental samples of the MMAS and the composite was 2.7 mm (inset in Figure 3). Because the Rayleigh range *z*_0_ = *πnω*^2^ / *λ* exceeded 10 cm, the calculation of Δ*α* and Δ*n* was performed using the formulae [40,41]:

(1) $$\left\{\begin{array}{l} \Delta \alpha \left(I\right)=\frac{2\sqrt{2}\Delta Τ\left(I\right)}{L},\\ \mathrm{\Delta n}\left(I\right)=\mathrm{\gamma I}=\frac{\lambda \Delta {Τ}_{\mathrm{pv}}\left(I\right)\times \left(\alpha +\Delta \alpha \left(I\right)\right)}{0.812\pi {\left(1-S\right)}^{0.27}\left(1-e^{-\left(\alpha +\Delta \alpha \left(I\right)\right)L}\right)}, \end{array}\right)$$

where Δ*T*(*I*) (Figure 4a) and Δ*T*_pv_(*I*) (Figure 5b) were the integral transmitted intensity and the normalized transmittance between the peak and valley at different radiation intensities, respectively; *λ* and *α* were the radiation wavelength and absorption coefficient (Figure 3), respectively, and *S* was the fraction of radiation transmitted by the aperture without the sample, which was 0.184.

The experimental curves *T*(*I*) and *T*_pv_(*I*), which contain information about Δ*T* and Δ*T*_pv_, showed that only the reverse saturable absorption of yellow radiation occurred in pure MMAS (Figure 4a). In contrast, the composite manifested the expected optical response: the shape of the experimental curves *T*(*I*) and *T*_pv_(*I*) indicated the saturable absorption of visible radiation in the composite and a negative change in its refractive index (Figure 5), and the values of Δ*T*(*I*) and Δ*T*_pv_(*I*) increased linearly with increasing intensities of blue (Figure 5a) and yellow (Figure 5b) radiation.

The approximation of *T*_pv_ based on the theoretical curves (solid lines in Figure 5) was performed using the equation [42]:

(2) $$T=1+\frac{2\left(-\rho x^{2}+2x-3\rho \right)}{\left(x^{2}+9\right)\left(x^{2}+1\right)}\Delta \Phi$$

where the coupling factor *ρ* = Δ*α* × λ / 4*π* × Δ*n* and the phase shift due to nonlinear refraction Δ*Φ* = 2*π* × Δ*n* × *L*_eff_ / *λ* had the following values: *ρ* = 0.09 and Δ*Φ* = −0.23 and −0.5 for blue radiation with intensities of 0.019 and 0.054 kW/cm^2^ and *ρ* = 0.05 and Δ*Φ* = −0.7 and −1.45 for yellow radiation with intensities of 0.04 and 0.093 kW/cm^2^.

## Discussion

**Figure 6:**
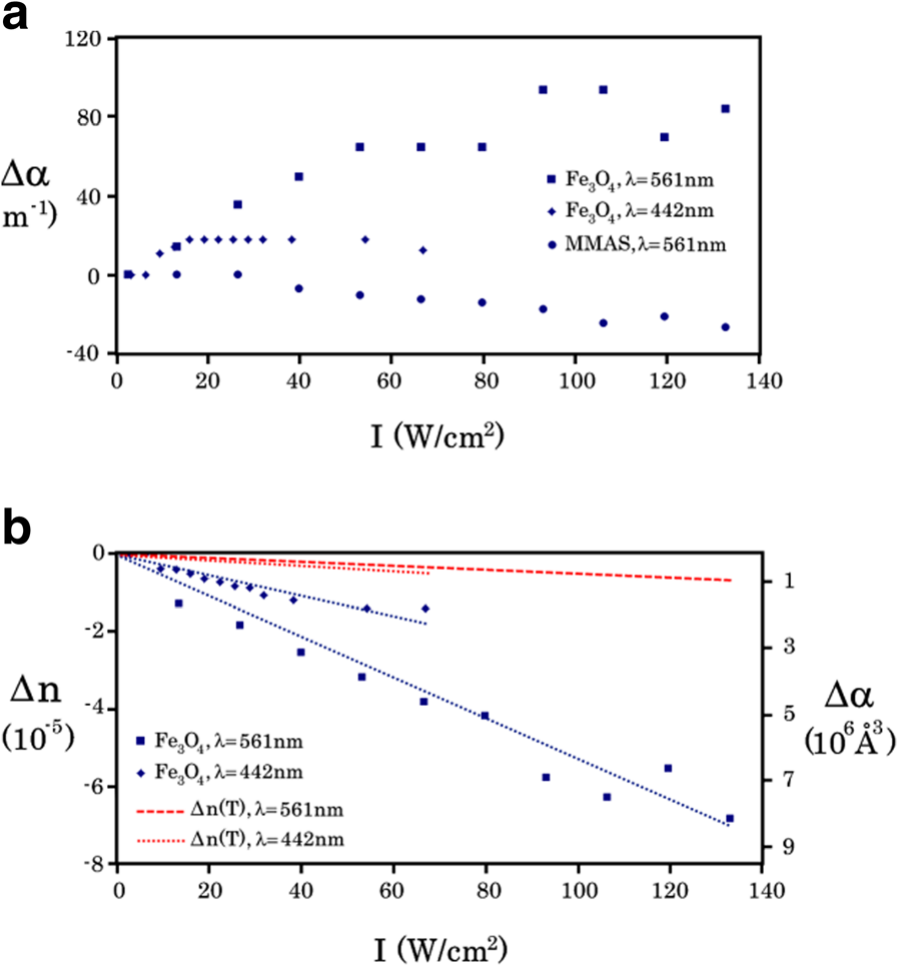
**The values of changes in the absorption coefficient, refractive index, and polarizability of Fe_3_O_4_ nanoparticles. (a)** The dependency of changes in the absorption coefficients Δ*α* of pure MMAS (circle) and Fe_3_O_4_ nanoparticle arrays (square and rhombus) on the intensity of radiation with wavelengths of 442 nm and 561 nm. **(b)** The dependency of changes in the refractive index Δ*n* and polarizability Δ*α* (Å^3^) of Fe_3_O_4_ nanoparticle arrays on the intensity of radiation with wavelengths of 442 nm (rhombus) and 561 nm (square); red dashed lines present the contribution of the thermal effect of cw radiation on the change in the refractive index (Equation 3), and blue dashed lines are theoretical approximations based on the approach of free carrier absorption (Equation 4).

The saturable absorption of visible radiation with intensities less than 0.14 kW/cm^2^ in the composite and the negative change in the refractive index were due to the presence of Fe_3_O_4_ nanoparticles since pure MMAS showed only the relatively weak reverse saturable absorption of yellow radiation. Therefore, the experimental data Δ*T*(*I*) and Δ*T*_pv_(*I*) obtained for the composite could be used to calculate the values of Δ*α*(*I*) and Δ*n*(*I*) for Fe_3_O_4_ nanoparticle arrays (Equation 1), and these values are listed in Figure 6.

Because the observed dependence of Δ*n* on the radiation intensity *I* (Figure 6b) for Fe_3_O_4_ nanoparticle arrays could be considered a linear function, it can be assumed that Δ*n* was caused by the thermal effect of the radiation. We estimated the contribution of this effect to the changes of the composite refractive index using the equation [43]:

(3) $$\Delta n_{\mathrm{therm}}=\frac{\Delta Ε\times \frac{\mathrm{dn}}{\mathrm{dT}}}{c_{\mathrm{hc}}\rho_{d}},$$

where *c*_hc_ was the MMAS heat capacity (0.7 J/g·K), *ρ*_d_ was the MMAS density (1.3 g/cm^3^), dn/dT was the MMAS thermo-optic coefficient (−10^−5^ K^−1^), and Δ*E* was the energy absorbed by the composite per unit volume per second. The thermal effect of cw low-intensity radiation on the change in the refractive index (red dashed lines in Figure 6b) was relatively small (not more than 20% for blue radiation and 8% for yellow radiation).

Generally, the possibility of a nonthermal optical response of the composite due to external optical radiation is associated with the polarization of Fe_3_O_4_ nanoparticles in the external field *E*. Nanoparticle polarization occurs at the spatial separation of positive and negative charges, i.e., at the electron transition to higher allowed energy states (quantum number l ≠ 0). These transitions should be accompanied by the absorption of external radiation. In our case, we observed the absorption of radiation with wavelengths of 380 to 650 nm (Figure 3). This absorption band consisted of three maxima (380, 480, and 650 nm), indicating the broadened quantum-size states for the electrons in Fe_3_O_4_ nanoparticles. Because the bandgap of magnetite is rather small (approximately 0.2 eV) [20-22], the conduction and valence bands of the nanoparticles should be coupled due to quantum-size effect [44]. Therefore, the transitions of Fe_3_O_4_ nanoparticle electrons to higher energy states by the action of photons with energies of 2.3 eV (*λ* = 561 nm) and 2.6 eV (*λ* = 442 nm) can be considered intraband transitions. In turn, these transitions result in changes in the refractive index of the media as follows [45-47]:

(4) $$\mathrm{\Delta n}\left(I\right)=-\frac{e^{2}\lambda^{2}}{8\pi^{2}c^{2}n_{0}\epsilon_{0}m_{e}}N_{e}$$

where *e* was the electron charge, *c* was the speed of light, *ϵ*_0_ was the electric constant, *m*_*e*_ was the electron mass, and *N*_*e*_ was the concentration of excited electrons, which depends on the number of photons in the beam or the radiation intensity *I*.

Using Equation 4 to approximate the experimentally observed behavior of Δ*n*(*I*) (Figure 6b, blue dashed lines), we estimated that the concentration of optically excited electrons in Fe_3_O_4_ nanoparticles was approximately 10^23^ m^−3^, being the radiation intensity of less than 0.14 kW/cm^2^.

The amplitude of the nanoparticle polarization is determined by **|E|** of the external field and the nanoparticle susceptibility (*χ*) or polarizability (*α*) measured in cubic angstrom. In turn, the change in the refractive index induced by the radiation is associated with the change in nanoparticle polarizability Δ*α* (Å^3^) by classical relations [48]. Therefore, we could calculate the values of Δ*α* (Å^3^) for Fe_3_O_4_ nanoparticle using the experimental values of Δ*n*(*I*) and the following equations (SI):

(5) $$\left\{\begin{array}{l} \epsilon =n^{2}\left(I\right)-k^{2}\left(I\right)=1+\chi \\ \Delta \chi =\Delta \alpha \left[{Å}^{3}\right]\cdot 10^{30}\cdot N\left[m^{-3}\right] \end{array}\right)$$

where *ϵ* was the real part of the dielectric constant, the composite refractive index *n*(*I*) = *n*_0_ + Δ*n*(*I*), and *n*_0_ was the refractive index of pure MMAS (approximately 1.5). The extinction coefficient *k* = *αλ* / 4*π* was significantly less than *n*(*I*) and could be ignored; *χ* was the nanoparticle susceptibility, and *N* was the nanoparticle concentration (approximately 2.3 × 10^19^ m^−3^). Therefore, the values of Δ*α* (Å^3^) for Fe_3_O_4_ nanoparticle were calculated using the formula Δ*α* (Å^3^) ≈ 2*n* × Δ*n*(*I*) × 10^30^ / *N* and are presented in Figure 6b.

The obtained values for the changes in nanoparticle polarizability are orders of magnitude greater than those for semiconductor nanoparticles and molecules [30,31] in extremely weak optical fields. In addition, the average nanoparticle volume was approximately 2.2 × 10^6^ Å^3^, and the maximum value of Δ*α* (Å^3^) was 9 × 10^6^ Å^3^. Thus, we can conclude that the nanoparticle polarization should be formed by several optical intraband transitions of nanoparticle electrons in weak optical fields.

## Conclusions

We used the developed co-precipitation method to synthesize spherical Fe_3_O_4_ nanoparticles covered with a monolayer of oleic acid that possessed a wide nonlinear absorption band of visible radiation 1.7 to 3.7 eV. The synthesized nanoparticles were dispersed in the optically transparent copolymer methyl methacrylate with styrene, and their optical properties were studied by optical spectroscopy and z-scan techniques. We report that the electric polarizability of Fe_3_O_4_ nanoparticles changes due to the effect of low-intensity visible radiation (*I* ≤ 0.2 kW/cm^2^; *λ* = 442 and 561 nm) and reaches a relatively high value of 10^7^ Å^3^. The change in polarizability is induced by the intraband phototransition of charge carriers and can be controlled by the intensity of the visible radiation used. This optical effect observed in magnetic nanoparticles may be employed to significantly improve the drug uptake properties of Fe_3_O_4_ nanoparticles.

## Abbreviations

Abs: Absorbance; Cw: Continuous wave; MMAS: Methyl methacrylate with styrene.

## Competing interests

The authors declare that they have no competing interests.

## Authors’ contributions

VM designed and performed the optical experiments (z-scan and spectroscopy), participated in the analysis and interpretation of data, and prepared the draft and final version of the manuscript. AN, VV, and VS designed and performed the chemical experiments, achieved that nanoparticle was covered with a monolayer of oleic acid, prepared the sections ‘Synthesis of nanoparticle’ and ‘Composite preparation’. YK and VD conceived of the study, participated in the analysis and interpretation of data, helped to draft the final version of the manuscript. All the authors read and approved the final manuscript.

## References

[B1] Gass J Poddar P Almand J Srinath S Srikanth H Superparamagnetic polymer nanocomposites with uniform Fe_3_O_4_ nanoparticle dispersions Adv Funct Mater 2006 8 71 75 10.1002/adfm.200500335

[B2] Wan J Tang G Qian Y Room temperature synthesis of single-crystal Fe_3_O_4_ nanoparticles with superparamagnetic property Appl Phys A 2007 8 261 264

[B3] Mürbe J Rechtenbach A Töpfer J Synthesis and physical characterization of magnetite nanoparticles for biomedical application Mater Chem Phys 2008 8 426 433 10.1016/j.matchemphys.2008.02.037

[B4] Hashimoto H Fujii T Nakanishi M Kusano Y Ikeda Y Takada J Synthesis and magnetic properties of magnetite-silicate nanocomposites derived from iron oxide of bacterial origin Mater Chem Phys 2012 8 1156 1161 10.1016/j.matchemphys.2012.08.070

[B5] Wang X Zhao Z Qu J Wang Z Qiu J Fabrication and characterization of magnetic Fe_3_O_4_-CNT composites J Phys Chem Sol 2010 8 673 676 10.1016/j.jpcs.2009.12.063

[B6] Xie J Chen K Lee HY Xu C Hsu AR Peng S Chen X Sun S Ultrasmall c(RGDyK)-coated Fe_3_O_4_ nanoparticles and their specific targeting to integrin α_v_β_3_-rich tumor cells J Am Chem Soc 2008 8 7542 7543 10.1021/ja802003h 18500805 PMC2542944

[B7] Mi C Zhang J Gao H Wu X Wang M Wu Y Di Y Xu Z Mao C Xu S Multifunctional nanocomposites of superparamagnetic (Fe3O4) and NIR-responsive rare earth-doped up-conversion fluorescent (NaYF4:Yb, Er) nanoparticles and their applications in biolabeling and fluorescent imaging of cancer cells Nanoscale 2010 8 1141 1148 10.1039/c0nr00102c 20648340 PMC3099179

[B8] Chen ZL Sun Y Huang P Yang XX Zhou XP Studies on preparation of photosensitizer loaded magnetic silica nanoparticles and their anti-tumor effects for targeting photodynamic therapy Nanoscale Res Lett 2009 8 400 408 10.1007/s11671-009-9254-5 20596490 PMC2893856

[B9] Yang C Wu J Hou Y Fe_3_O_4_ nanostructures: synthesis, growth mechanisms, properties and application Chem Commun 2011 8 5130 5141 10.1039/c0cc05862a 21384025

[B10] Wang X Zhang R Wu C Dai Y Song M Gutmann S Gao F Lu G Li J Li X Guan Z Fu D Chen B The application of Fe_3_O_4_ nanoparticles in cancer research: a new strategy to inhibit drug resistance J Biomed Mater Res A 2007 8 4 852 860 10.1002/jbm.a.30901 17072850

[B11] Gong P Li H He X Wang K Hu J Tan W Zhang S Yang X Preparation and antibacterial activity of Fe_3_O_4_@Ag nanoparticles Nanotechnology 2007 8 1 7 285604

[B12] Liu X Hu Q Fang Z Wu Q Xie Q Carboxyl enriched monodisperse porous Fe_3_O_4_ nanoparticles with extraordinary sustained-release property Langmuir Lett 2009 8 13 7244 7248 10.1021/la901407d 19507833

[B13] Covaliu CI Berger D Matei C Diamandescu L Vasile E Cristea C Ionita V Iovu H Magnetic nanoparticles coated with polysaccharide polymers for potential biomedical applications J Nanopart Res 2011 8 6169 6180 10.1007/s11051-011-0452-6

[B14] Wu KT Kuo PC Yao YD Tsai EH Magnetic and optical properties of Fe_3_O_4_ nanoparticle ferrofluids prepared by coprecipitation technique IEEE Trans Magn 2001 8 4 2651 2653 10.1109/20.951263

[B15] Narsinga Rao G Yao YD Chen YL Wu KT Chen JW Particle size and magnetic field-induced optical properties of magnetic fluid nanoparticles Phys Rev E 2005 8 1 6 10.1103/PhysRevE.72.031408 16241436

[B16] Liu T Chen X Di Z Zhang J Tunable magneto-optical wavelength filter of long-period fiber grating with magnetic fluids Appl Phys Lett 2007 8 121116 10.1063/1.2787970

[B17] Li J Liu X Lin Y Bai L Li Q Chen X Field modulation of light transmission through ferrofluid film Appl Phys Lett 2007 8 1 3 253108

[B18] Chieh JJ Hong CY Yang SY Horng HE Yang HC Study on magnetic fluid optical fiber devices for optical logic operations by characteristics of superparamagnetic nanoparticles and magnetic fluids J Nanopart Res 2010 8 293 300 10.1007/s11051-009-9613-2

[B19] Xia SH Wang J Lu ZX Zhang F Birefringence and magneto-optical properties in oleic acid coated Fe_3_O_4_ nanoparticles: application for optical switch Int J Nanoscience 2011 8 3 515 520 10.1142/S0219581X11008289

[B20] Balberg I Pankove JI Optical measurements on magnetite single crystals Phys Rev Lett 1971 8 9 596 599 10.1103/PhysRevLett.27.596

[B21] Park JH Tjeng LH Allen JW Metcalf P Chen CT Single-particle gap above the Verwey transition in Fe_3_O_4_ Phys Rev B 1997 8 19 813 817

[B22] Jordan K Cazacu A Manai G Ceballos SF Murphy S Shvets IV Scanning tunneling spectroscopy study of the electronic structure of Fe_3_O_4_ surface Phys Rev B 2006 8 1 6 085416

[B23] Buchenau U Müller I Optical properties of magnetite Solid State Commun 1972 8 1291 1293 10.1016/0038-1098(72)90845-9

[B24] Muret P Optical absorption in polycrystalline thin films of magnetite at room temperature Solid State Commun 1974 8 1119 1122 10.1016/0038-1098(74)90286-5

[B25] Schlegel A Alvarado SF Wachter P Optical properties of magnetite (Fe_3_O_4_) J Phys C: Solid State Phys 1979 8 1157 1164 10.1088/0022-3719/12/6/027

[B26] Fontijn WFJ van der Zaag PJ Devillers MAC Brabers VAM Metselaar R Optical and magneto-optical polar Kerr spectra of Fe_3_O_4_ and Mg^2+^ - or Al^3+^-substituted Fe_3_O_4_ Phys Rev B 1997 8 9 5432 5442 10.1103/PhysRevB.56.5432

[B27] Yasumori A Matsumoto H Hayashi S Okada K Magneto-optical properties of silica gel containing magnetite fine particles J Sol–gel Sci Tech 2000 8 249 258 10.1023/A:100870010741523844339

[B28] Barnakov YA Scott BL Golub V Kelley L Reddy V Stokes KL Spectral dependence of Faraday rotation in magnetite-polymer nanocomposites J Phys Chem Solids 2004 8 1005 1010 10.1016/j.jpcs.2003.10.070

[B29] Roychowdhury A Pati SP Mishra AK Kumar S Das D Magnetically addressable fluorescent Fe_3_O_4_/ZnO nanocomposites: structural, optical and magnetization studies J Phys Chem Solids 2013 8 811 818 10.1016/j.jpcs.2013.01.012

[B30] Evlyukhin AB Reinhardt C Seidel A Luk’yanchuk BS Chichkov BN Optical response features of Si-nanoparticle arrays Phys Rev B 2010 8 4 1 12 045404

[B31] Marenich AV Cramer CJ Truhlar DG Reduced and quenched polarizabilities of interior atoms in molecules Chem Sci 2013 8 2349 2356 10.1039/c3sc50242b

[B32] Kang YS Risbud S Rabolt JF Stroeve P Synthesis and characterization of nanometer-size Fe_3_O_4_ and γ- Fe_3_O_4_ particles Chem Mater 1996 8 2209 2211 10.1021/cm960157j

[B33] Chen L Yang WJ Yang CZ Preparation of nanoscale iron and Fe_3_O_4_ powders in a polymer matrix J Mater Sci 1997 8 3571 3575 10.1023/A:1018613926326

[B34] Long Y Chen Z Duvali JL Zhang Z Wan M Electrical and magnetic properties of polyaniline/Fe_3_O_4_ nanostructures Physica B 2005 8 121 130 10.1016/j.physb.2005.09.009

[B35] Banert T Peuker UA Preparation of highly filled super-paramagnetic PMMA-magnetite nanocomposites using the solution method J Mater Sci 2006 8 3051 3056 10.1007/s10853-006-6976-y

[B36] Li D Jiang D Chen M Xie J Wu Y Dang S Zhang J An easy fabrication of monodisperse oleic acid-coated Fe_3_O_4_ nanoparticles Mater Lett 2010 8 2462 2464 10.1016/j.matlet.2010.08.025

[B37] Gnanaprakash G Mahadevan S Jayakumar T Kalyanasundaram P Philip J Raj B Effect of initial pH and temperature of iron salt solutions on formation of magnetite nanoparticles Mater Chem Phys 2007 8 168 175 10.1016/j.matchemphys.2007.02.011

[B38] Tural B Özkan N Volkan M Preparation and characterization of polymer coated superparamagnetic magnetite nanoparticle agglomerates J Phys Chem Solids 2009 8 860 866 10.1016/j.jpcs.2009.04.007

[B39] Lan Q Liu C Yang F Liu S Xu J Sun D Synthesis of bilayer oleic acid-coated Fe_3_O_4_ nanoparticles and their application in pH-responsive Pickering emulsions J Coll Interf Sci 2007 8 260 269 10.1016/j.jcis.2007.01.081 17382339

[B40] Milichko VA Dzyuba VP Kulchin YN Unusual nonlinear optical properties of SiO_2_ nanocomposite in weak optical fields Appl Phys A 2013 8 1 319 322

[B41] Sheik-Bahae M Said AA Wei TH Hagan DJ Van Stryland EW Sensitive measurement of optical nonlinearities using a single beam IEEE J Quantum Electron 1990 8 4 760 769 10.1109/3.53394

[B42] Liu X Guo S Wang H Hou L Theoretical study on the closed-aperture Z-scan curves in the materials with nonlinear refraction and strong nonlinear absorption Opt Commun 2001 8 431 437 10.1016/S0030-4018(01)01406-7

[B43] Ganeev RA Ryasnyansky AI Stepanov AL Usmanov T Nonlinear optical response of silver and copper nanoparticles in the near-ultraviolet spectral range Phys Sol State 2004 8 2 351 356 10.1134/1.1649436

[B44] AlL E Rosen M Quantum size level structure of narrow-gap semiconductor nanocrystals: effect of band coupling Phys Rev B 1998 8 11 7120 7135 10.1103/PhysRevB.58.7120

[B45] Bennett BR Soref RA Del Alamo J Carrier-induced change in refractive index of InP, GaAs, and InGaAsP IEEE J Quantum Electron 1990 8 1 113 122 10.1109/3.44924

[B46] Veselago VG The electrodynamics of substances with simultaneously negative values of *ϵ* and *μ* Physics-Uspekhi 1968 8 509 514 10.1070/PU1968v010n04ABEH003699

[B47] Yu ZG Krishnamurthy S Guha S Photoexcited-carrier-induced refractive index change in small bandgap semiconductors J Opt Soc Am B 2006 8 11 2356 2360 10.1364/JOSAB.23.002356

[B48] Akhmanov A Nikitin SY Physical Optics 1997 Oxford: Oxford University Press

